# Supplementary material for: Stable isotopes show that earthquakes enhance permeability and release water from mountains
Source: Nat Commun. 2020 Jun 2;11:2776. doi: 10.1038/s41467-020-16604-y (PMC7265347; doi:10.1038/s41467-020-16604-y)
Supplement: Supplementary file 1 — Supplementary Information [file 41467_2020_16604_MOESM1_ESM.pdf]

Supplementary Information for "Stable isotopes show that earthquakes enhance permeability and release water from mountains" by Hosono et al.

Takahiro Hosono[1,2]*, Chisato Yamada[3], Michael Manga[4], Chi-Yuen Wang[4], Masaharu Tanimizu[5]

[1] Faculty of Advanced Science and Technology, Kumamoto University, 2-39-1 Kurokami, Kumamoto 860-8555, Japan

[2] International Research Organization for Advanced Science and Technology, Kumamoto University, 2-39-1 Kurokami, Kumamoto 860-8555, Japan

[3] Department of Earth Science, Faculty of Science, Kumamoto University, 2-39-1 Kurokami, Kumamoto 860-8555, Japan

[4] Department of Earth and Planetary Science, University of California, Berkeley McCone Hall, Berkeley, CA, United States

[5] School of Science and Technology, Kwansei Gakuin University, 2-1 Gakuen, Sanda, 669-1337, Japan

*Correspondence to: hosono@kumamoto-u.ac.jp (Takahiro Hosono)

This file includes:

Supplementary Figures 1-10
Supplementary Tables 1-3

Supplementary Figures

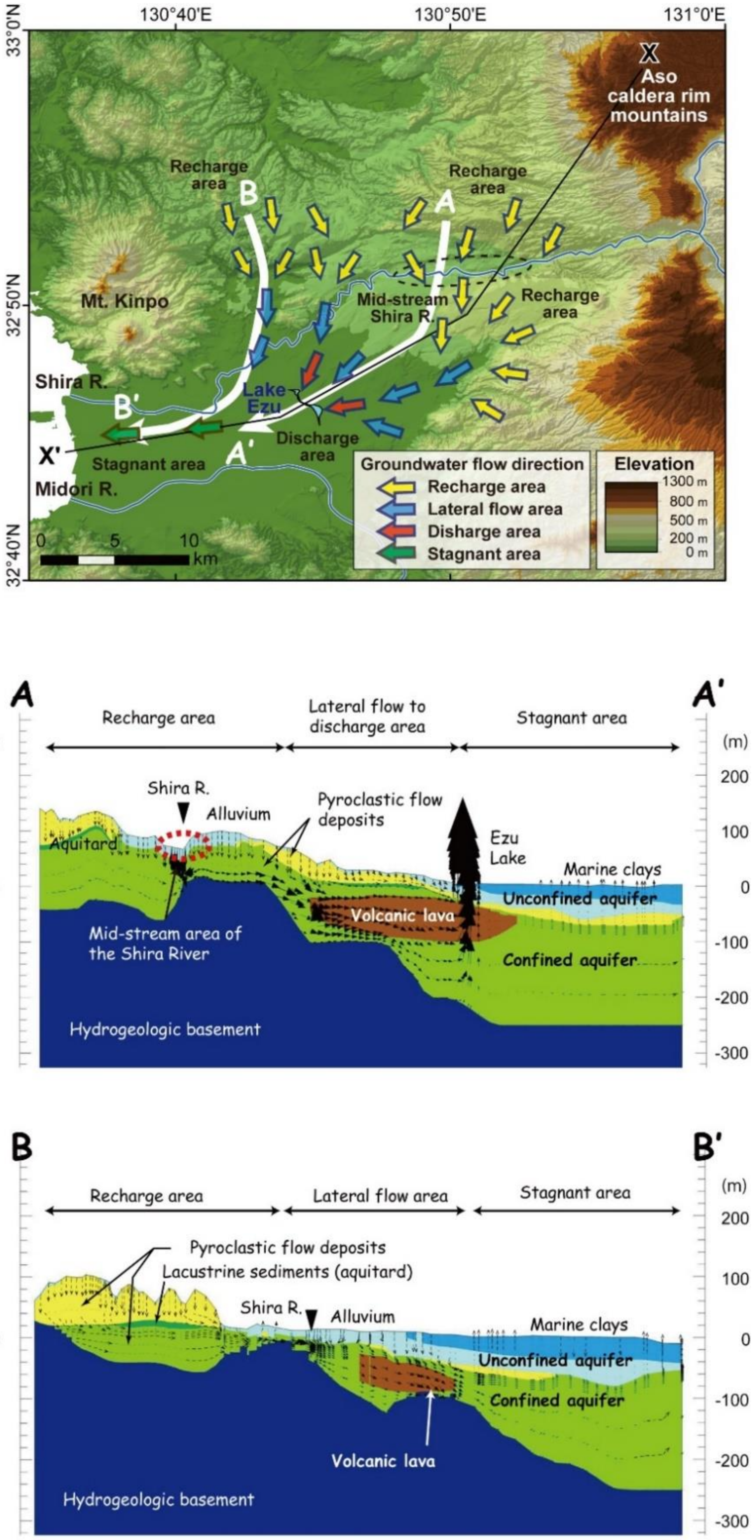

Supplementary Figure 1. Cross sectional maps. Simplified hydrogeological cross section along A-A’ and B-B’ after refs. 1 and 2. Both flow lines are shown in uppermost figure. Black arrows in the cross-sections show the groundwater flow direction and relative magnitude calculated by the GETFLOWS simulator[3].

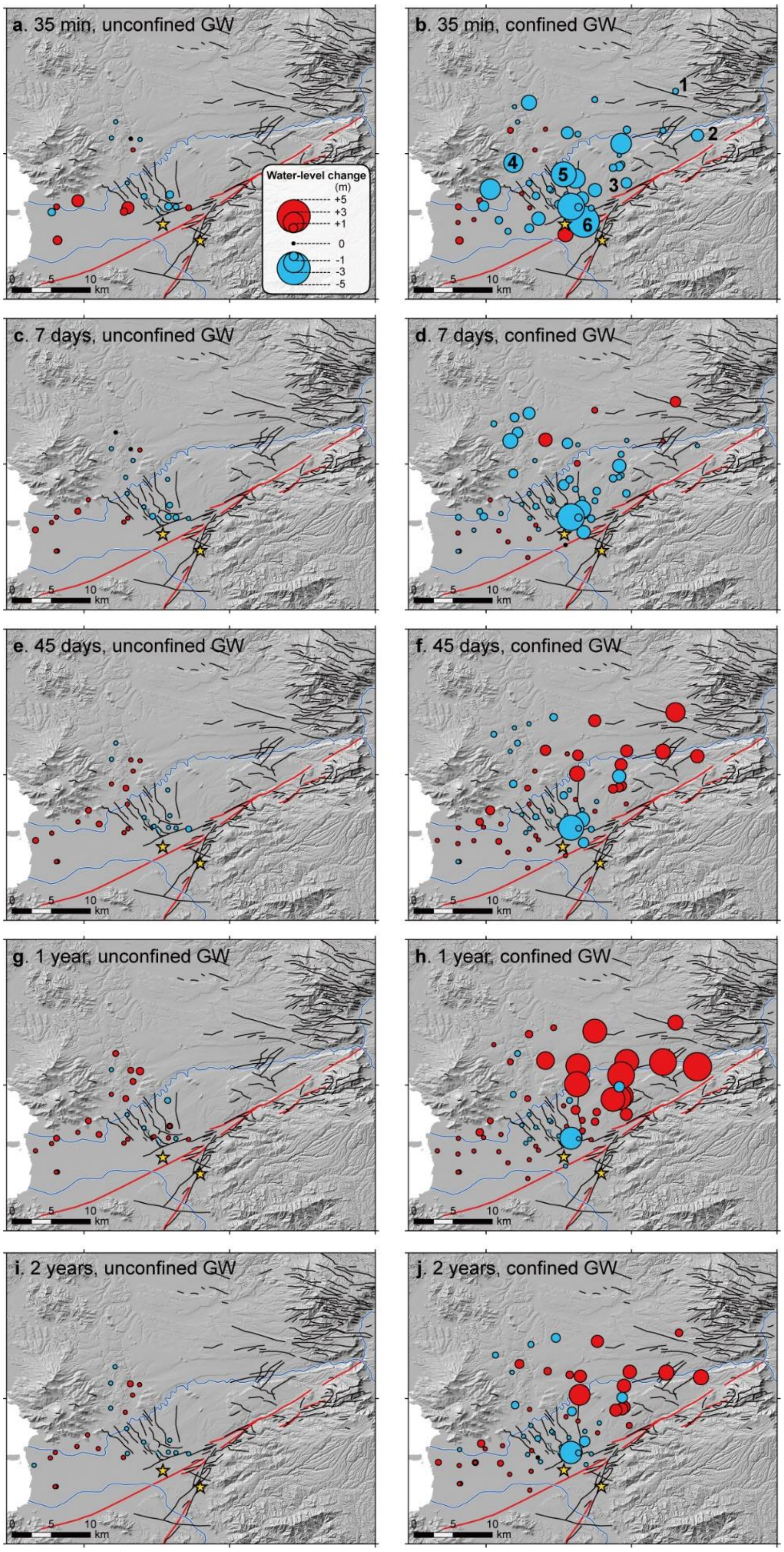

Supplementary Figure 2. Coseismic changes in groundwater levels. (**a-j**) Relative water level changes comparing water levels before the start of the Kumamoto earthquake sequence (26 minutes

before the foreshock, 21:00 JST, 14 April 2016) and 35 minutes, 7 days, 45 days, 1 year, and 2 years after the main shock (01:25 JST, 16 April 2016) separately shown for unconfined and confined aquifers, respectively. The earthquake epicenters and fault systems are shown in Fig. 1b. Numbers (1 to 6) labeled in or beside the symbols in b correspond the well numbers shown in Supplementary Fig. 3. The panels are modified after ref. 1.

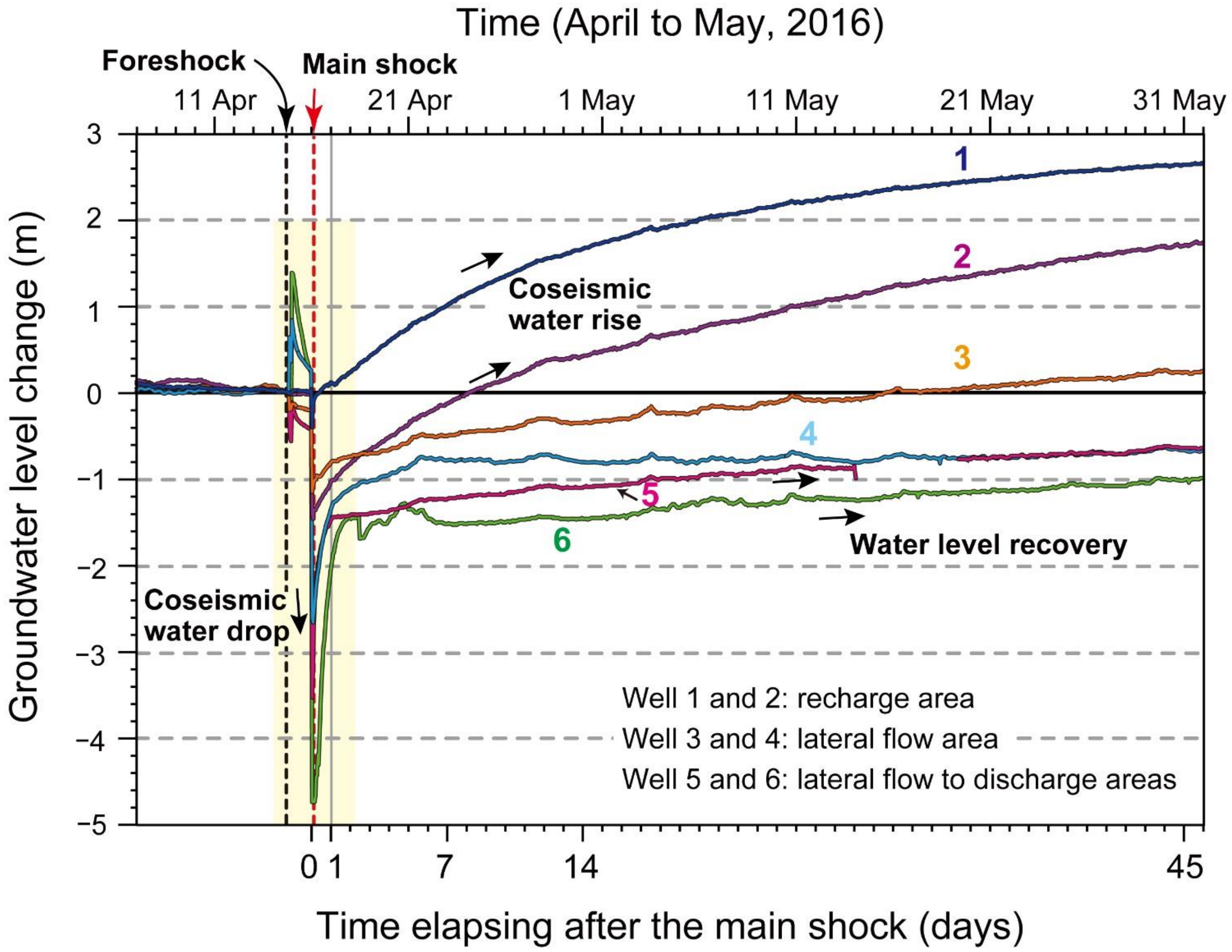

Supplementary Figure 3. Water level change time series. Post-earthquake water level changes relative to the water levels before the earthquake (21:00 JST, 14 April 2016) during the first 45 days after the main shock of the 2016 Kumamoto earthquake for selected wells for the confined aquifer are shown using hourly monitored digital data (modified after ref. 1). The numbers in the figure (1 to 6) correspond the well numbers shown in Supplementary Fig. 2b.

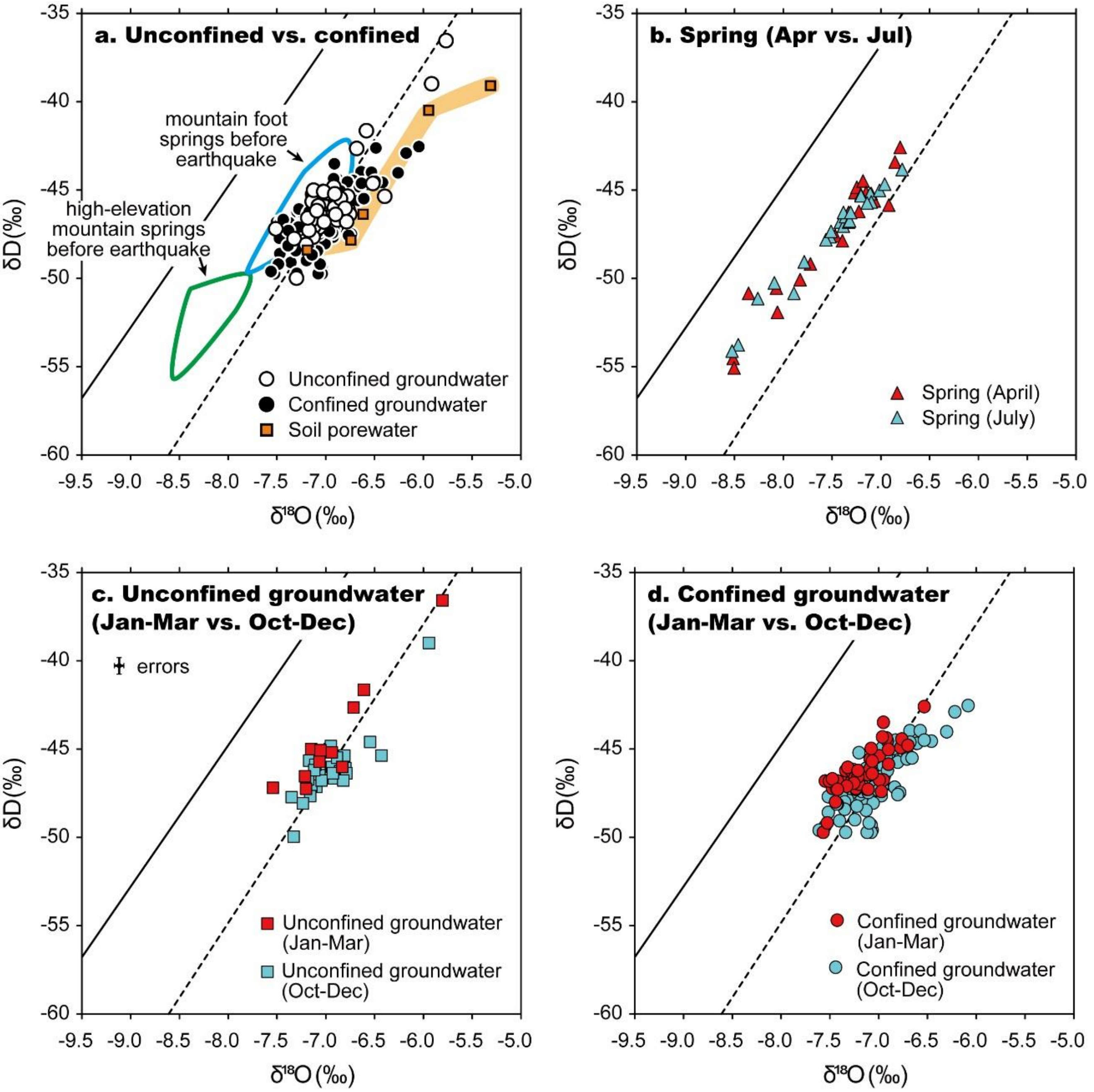

Supplementary Figure 4. Isotopic compositions of water samples collected before the earthquake. (**a**) Isotopic comparison between unconfined and confined groundwaters. (**b**) Compositional variations between different sampling months for mountain springs. (**c,d**) Comparison of stable isotopic compositions for the water samples collected at different months from unconfined and confined groundwaters, respectively. The two local meteoric lines are the same as in Fig. 2. Errors (analytical precision) are shown panel c.

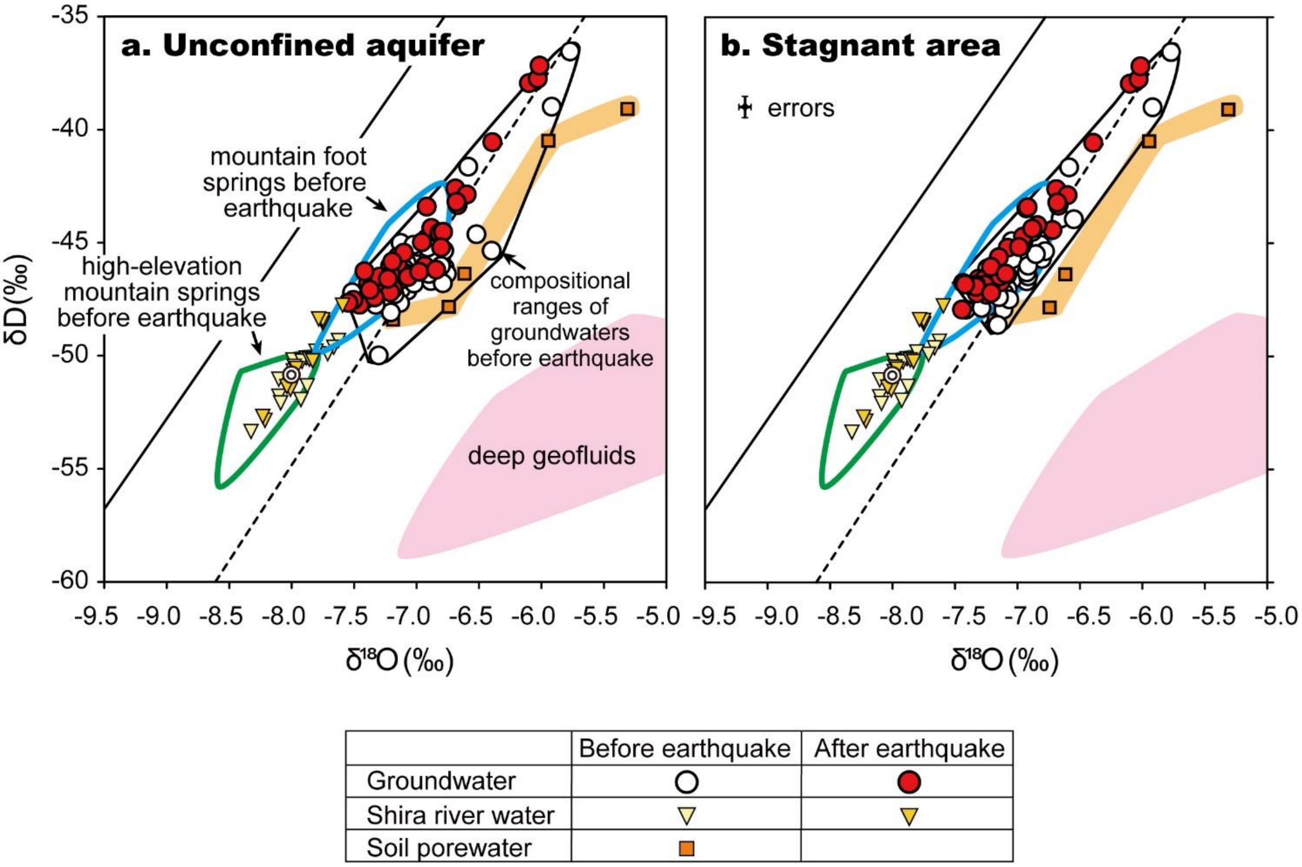

Supplementary Figure 5. Coseismic changes in stable isotope ratios. (**a**) Compositional changes between before (November, 2009 to November, 2011) and after (June, 2016 to December, 2017) the main shock for groundwaters from unconfined aquifers. (**b**) Compositional changes of groundwaters for stagnant area. Samples from all seasons are plotted in the figures. Plot symbols and two local meteoric lines are the same as in Fig. 3b-d. Errors are shown in panel b.

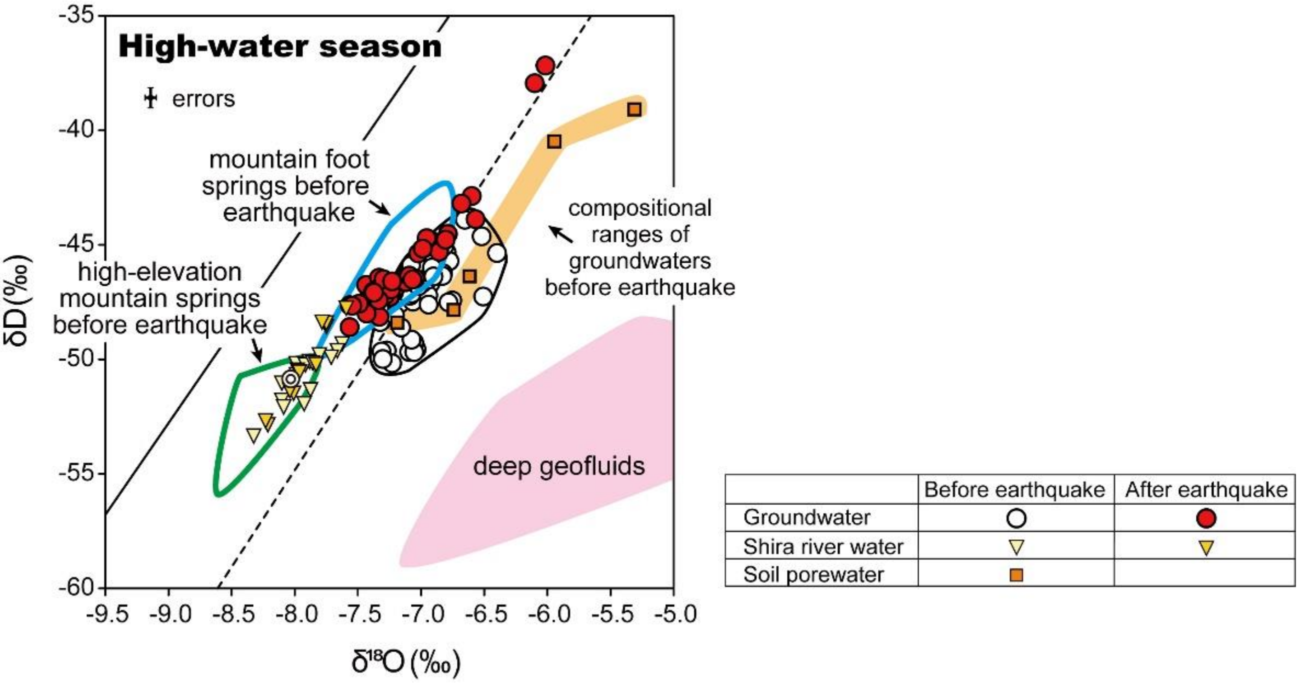

Supplementary Figure 6. Coseismic changes in stable isotope ratios of groundwaters in same season. Compositional changes between before (years 2009, 2010 and 2011) and after (year 2016 and 2017) the main shock for the samples collected during October and November for both unconfined and confined aquifers. Plot symbols and two local meteoric lines are the same as in Fig. 3b-d.

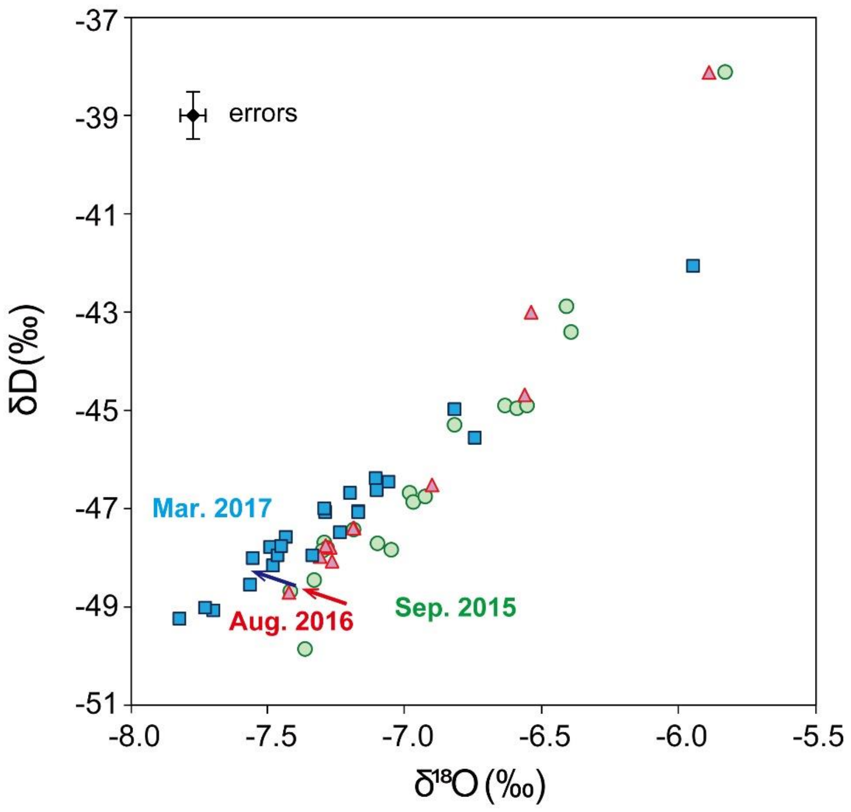

Supplementary Figure 7. Coseismic changes in stable isotope ratios of groundwaters collected from cross-check sampling surveys. The isotopic compositions for groundwater samples collected during September 2015 (green circle), August 2016 (red triangle), and March 2017 (blue square) are compared. Sampling locations are shown in Supplementary Fig. 10.

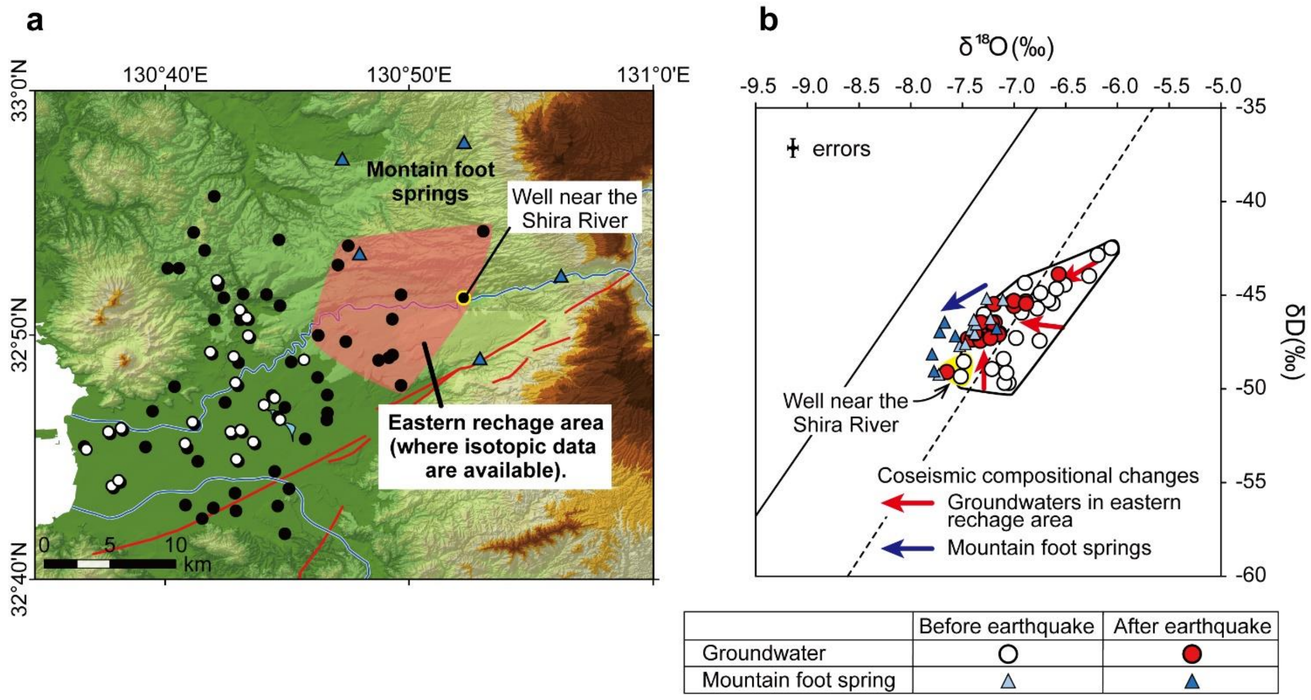

Supplementary Figure 8. Isotopic evidence of permeability enhancement. (**a**) Map showing the locations of samples for groundwaters in the eastern recharge area and mountain foot spring waters used for isotopic comparisons in panel b. (**b**) Oxygen and hydrogen stable isotope ratios for groundwaters in eastern recharge area and mountain foot spring waters in vicinity of eastern recharge area where the most significant water level rise was observed (Supplementary Fig. 2). Plot symbols and two local meteoric lines are the same as in Fig. 3b-d. Groundwater samples after the earthquake are shown in red, while those before the earthquake are shown in white. Spring water samples (blue triangles) obtained after the earthquake are shown in darker colors than samples from before the earthquake. Note that isotope ratios of groundwater samples with variable compositions changed toward the narrow compositional field of mountain spring waters except for one well near the Shira River which records mixing of river water both before and after the earthquake.

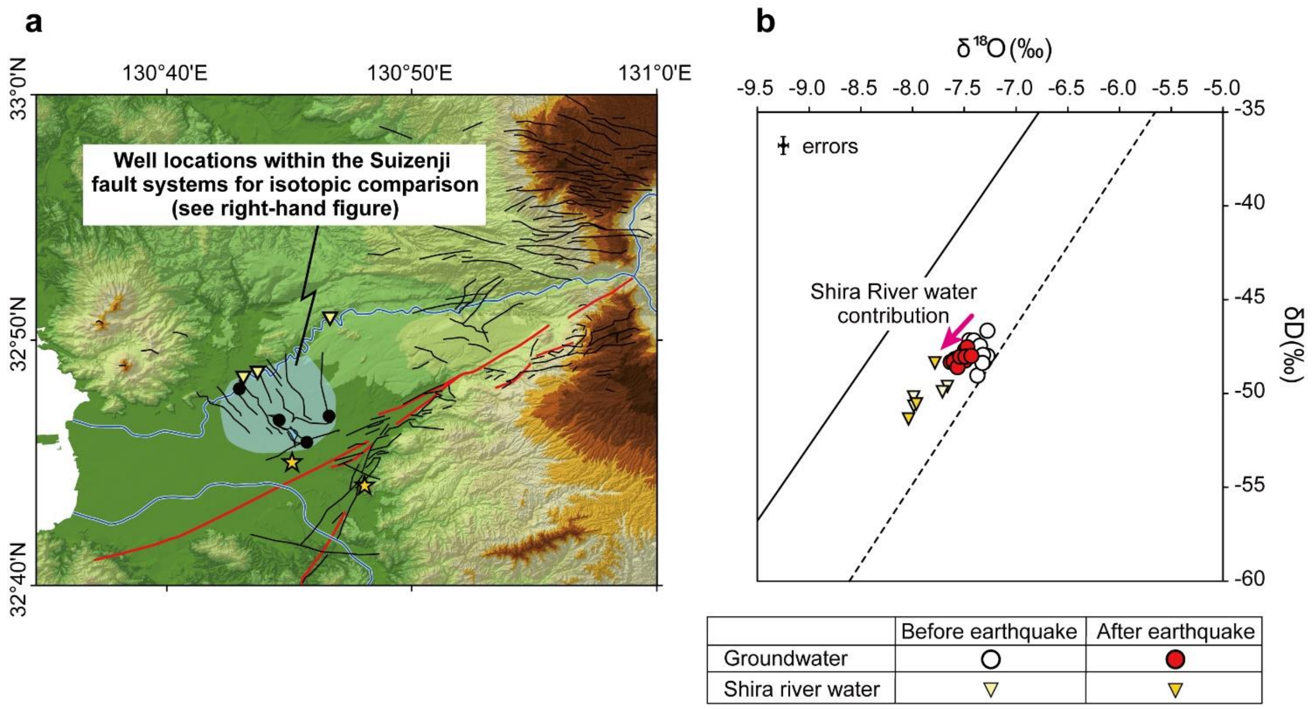

Supplementary Figure 9. Isotopic evidence of surface water contribution. (**a**) Map showing the locations of samples for groundwaters in the Suizenji fault systems (area shown in light blue) and Shira River waters used for isotopic comparison in panel b. Locations for the groundwaters mixed with Shira River water are shown with circles. (**b**) Oxygen and hydrogen stable isotope ratios for groundwaters in the Suizenji fault systems for selected wells. Plot symbols and two local meteoric lines are the same as in Fig. 3b-d. Groundwater samples after the earthquake are shown in red, while ones before the earthquake are shown in white. River water samples (yellow triangles) obtained after the earthquake are shown in darker colors than samples from before the earthquake.

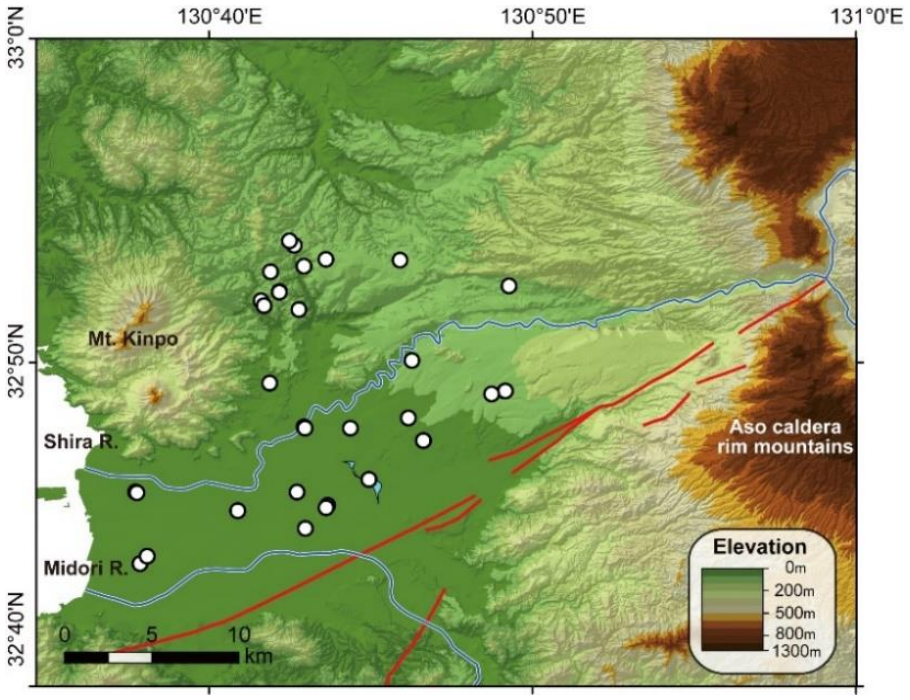

Supplementary Figure 10. Sampling locations of groundwaters for cross-check sampling surveys. The groundwater samples were collected during September 2015, August 2016, and March 2017. See Supplementary Fig. 7 for the data plots.

Supplementary Tables

Supplementary Table 1a-g. Properties and oxygen and hydrogen stable isotope ratios (δD and $\delta^{18}O$) for water samples used in this study: (**a**) river waters, (**b**) mountain foot spring waters, (**c**) high-elevation mountain spring waters, (**d**) unconfined groundwaters, (**e**) confined groundwaters, (**f**) hot spring waters, and (**g**) mountain water obtained from tunnel.

Supplementary Table 1a. Properties, chemistry, and oxygen and hydrogen stable isotope ratios (δD and $\delta^{18}O$) for river water samples used in this study.

Before the earthquake

Sample ID	Latitude	Longitude	Sampling Day	δD_{H2O} (‰)	$\delta^{18}O_{H2O}$ (‰)
R1	32.76991	130.64679	2011/7/21	-49.3	-7.63
R1	32.76991	130.64679	2011/4/21	-50.1	-7.88
R2	32.81128	130.72869	2011/7/26	-49.6	-7.66
R2	32.81128	130.72869	2011/4/21	-50.7	-7.99
R3	32.84814	130.77791	2011/7/26	-49.9	-7.71
R3	32.84814	130.77791	2011/4/21	-50.2	-7.99
R4	32.85647	130.86816	2011/4/20	-51.0	-8.10
R5	32.85855	130.87583	2011/7/26	-49.8	-7.81
R5	32.85855	130.87583	2011/4/20	-50.2	-7.92
R6	32.87214	130.93791	2011/7/22	-50.2	-7.86
R6	32.87214	130.93791	2011/4/20	-51.8	-8.10
R7	32.82256	131.04814	2011/7/22	-52.1	-8.09
R7	32.82256	131.04814	2011/4/20	-53.4	-8.33
R8	32.96641	131.13261	2011/7/22	-51.9	-7.93
R8	32.96641	131.13261	2011/4/20	-51.3	-7.88

After the earthquake

Sample ID	Latitude	Longitude	Sampling Day	δD_{H2O} (‰)	$\delta^{18}O_{H2O}$ (‰)
RW1	32.77169	130.68650	2016/8/8	-50.5	-7.98
RW1	32.77169	130.68650	2016/10/31	-51.5	-8.01
RW1	32.77169	130.68650	2017/4/20	-48.6	-7.75
RW2	32.77636	130.68775	2016/8/8	-48.4	-7.75
RW2	32.77636	130.68775	2016/10/31	-50.2	-7.83
RW2	32.77636	130.68775	2017/4/20	-47.8	-7.59
RW3	32.80778	130.71897	2016/8/8	-50.6	-7.96
RW3	32.80778	130.71897	2016/10/31	-51.4	-8.04
RW3	32.80778	130.71897	2017/4/20	-48.4	-7.79
RW4	32.85839	131.01133	2016/10/26	-52.9	-8.21

RW4	32.85839	131.01133	2017/4/30	-52.7	-8.23

Supplementary Table 1b. Properties, chemistry, and oxygen and hydrogen stable isotope ratios (δD and $\delta^{18}O$) for mountain foot spring water samples used in this study (after ref. 4).

Before the earthquake

Sample ID	Longitude	Latitude	Sampling Day	δD_{H2O} (‰)	$\delta^{18}O_{H2O}$ (‰)
S1	32.96384	130.87090	2011/7/20	-47.7	-7.53
S1	32.96384	130.87090	2011/4/18	-49.2	-7.73
S2	32.87260	130.93711	2011/7/22	-45.2	-7.11
S2	32.87260	130.93711	2011/4/20	-46.3	-7.23
S3	32.81627	130.88169	2011/7/20	-46.8	-7.42
S3	32.81627	130.88169	2011/4/19	-47.6	-7.47
S4	32.88778	130.79955	2011/7/20	-47.1	-7.38
S5	33.01249	130.83619	2011/7/20	-46.5	-7.37
S5	33.01249	130.83619	2011/4/18	-45.2	-7.26
S6	32.95253	130.78771	2011/7/20	-46.3	-7.39
S7	32.74142	130.83978	2011/7/21	-46.8	-7.33
S7	32.74142	130.83978	2011/4/21	-47.9	-7.40
S8	32.67964	130.76495	2011/7/21	-43.9	-6.79
S8	32.67964	130.76495	2011/4/19	-42.6	-6.81
S9	32.90834	130.71180	2011/7/19	-45.7	-7.10
S9	32.90834	130.71180	2011/4/15	-45.7	-7.07
S10	32.83006	130.63390	2011/7/19	-47.8	-7.56
S10	32.83006	130.63390	2011/4/15	-44.9	-7.25
S11	32.81255	130.67964	2011/7/19	-45.1	-7.02
S11	32.81255	130.67964	2011/4/15	-43.4	-6.85
S12	32.84704	130.72332	2011/7/19	-44.7	-6.96
S12	32.84704	130.72332	2011/4/15	-45.9	-6.92
S13	32.88313	130.65556	2011/7/19	-46.8	-7.33
S14	32.78265	130.62367	2011/7/19	-47.4	-7.51
S14	32.78265	130.62367	2011/4/15	-44.5	-7.19
S15	32.84675	130.58422	2011/7/19	-45.3	-7.20
S15	32.84675	130.58422	2011/4/15	-45.4	-7.16
S16	32.82965	130.59088	2011/7/19	-45.4	-7.20
S16	32.82965	130.59088	2011/4/15	-45.2	-7.15
S17	32.78210	130.74068	2011/7/21	-45.8	-7.14

S17	32.78210	130.74068	2011/4/21	-45.2	-7.10
S18	32.76995	130.77078	2011/7/21	-46.3	-7.31
S18	32.76995	130.77078	2011/4/21	-46.3	-7.33

After the earthquake

Sample ID	Latitude	Longitude	Sampling Day	δD_{H2O} (‰)	$\delta^{18}O_{H2O}$ (‰)
S1	32.96384	130.87090	2017/6/4	-49.1	-7.78
S2	32.87162	130.93078	2017/6/6	-47.2	-7.57
S3	32.81394	130.87259	2017/6/5	-48.1	-7.79
S4	32.95253	130.78771	2017/6/4	-47.0	-7.72
S5	33.01364	130.83619	2017/6/4	-46.4	-7.67
S6	32.88783	130.79954	2017/6/4	-46.8	-7.17
S7	32.75610	130.77875	2017/6/5	-48.2	-7.74
S8	32.68209	130.76731	2017/5/17	-46.0	-7.65
S9	32.90834	130.71180	2017/5/28	-46.3	-7.19
S10	32.83006	130.63390	2017/5/28	-46.3	-7.37
S11	32.84704	130.72332	2017/5/28	-46.2	-7.01
S12	32.81255	130.67964	2017/5/28	-45.2	-7.10
S13	32.88316	130.65587	2017/5/28	-47.0	-7.33
S14	32.78265	130.62367	2017/5/28	-46.6	-7.44
S15	32.82965	130.59088	2017/5/28	-46.3	-7.32
S16	32.84675	130.58422	2017/5/28	-46.6	-7.37
S17	32.78210	130.74068	2017/6/5	-46.5	-7.50
S18	32.76995	130.77078	2017/6/5	-47.9	-7.73

Supplementary Table 1c. Properties, chemistry, and oxygen and hydrogen stable isotope ratios (δD and $\delta^{18}O$) for high-elevation mountain spring water samples used in this study (after ref. 4).

Before the earthquake

Sample ID	Longitude	Latitude	Sampling Day	δD_{H2O} (‰)	$\delta^{18}O_{H2O}$ (‰)
HS1	32.98042	131.08104	2011/7/22	-49.1	-7.79
HS1	32.98042	131.08104	2011/4/20	-50.1	-7.84
HS2	32.82366	131.08706	2011/7/22	-53.8	-8.47
HS2	32.82366	131.08706	2011/4/20	-54.6	-8.52
HS3	32.83338	131.04139	2011/7/22	-50.9	-7.90
HS3	32.83338	131.04139	2011/4/20	-52.0	-8.06
HS4	32.93122	130.99432	2011/7/22	-50.3	-8.10
HS4	32.93122	130.99432	2011/4/20	-50.6	-8.07

HS5	32.95198	131.09475	2011/7/22	-54.2	-8.53
HS5	32.95198	131.09475	2011/4/20	-55.2	-8.51
HS6	32.77447	130.93776	2011/7/21	-51.2	-8.26
HS6	32.77447	130.93776	2011/4/19	-50.9	-8.36

After the earthquake

Sample ID	Latitude	Longitude	Sampling Day	δD_{H2O} (‰)	$\delta^{18}O_{H2O}$ (‰)
HS1	32.98042	131.08104	2017/6/6	-50.2	-8.08
HS2	32.82366	131.08706	2017/6/6	-54.3	-8.69
HS2	32.82366	131.08706	2016/10/26	-54.3	-8.63
HS2	32.82366	131.08706	2017/4/30	-54.7	-8.51
HS3	32.83345	131.04104	2017/6/6	-52.7	-8.36
HS4	32.93113	130.99156	2017/6/6	-51.0	-8.43
HS5	32.95198	131.09475	2017/6/6	-54.8	-8.76
HS5	32.95198	131.09475	2016/10/26	-54.9	-8.66
HS5	32.95198	131.09475	2017/4/30	-55.3	-8.50
HS6	32.77447	130.93776	2016/10/26	-52.2	-8.34
HS6	32.77447	130.93776	2017/3/30	-51.8	-8.38
HS6	32.77447	130.93776	2017/6/5	-51.8	-8.61

Supplementary Table 1d. Properties, chemistry, and oxygen and hydrogen stable isotope ratios (δD and $\delta^{18}O$) for unconfined groundwater samples used in this study.

Before the earthquake

Sample ID	Longitude	Latitude	Screen Depth (m)	Well Depth (m)	Sampling Day	δD_{H2O} (‰)	$\delta^{18}O_{H2O}$ (‰)
UGW1	32.72976	130.63116	4.5-10	10.0	2011/2/14	-36.5	-5.77
UGW2	32.73361	130.63583	5.0-10	10.0	2009/11/6	-39.0	-5.92
UGW3	32.74793	130.71634	24.7-35.7	41.2	2010/10/20	-46.5	-7.03
UGW3	32.74793	130.71634	24.7-35.7	41.2	2010/12/27	-45.4	-7.11
UGW3	32.74793	130.71634	24.7-35.7	41.2	2011/11/1	-45.0	-6.93
UGW4	32.75461	130.61392	7.0-11	15.0	2009/11/6	-45.7	-6.80
UGW5	32.75868	130.68128	31.7-42.7	45.7	2010/10/20	-46.3	-6.92
UGW5	32.75868	130.68128	31.7-42.7	45.7	2011/2/14	-45.7	-7.04
UGW5	32.75868	130.68128	31.7-42.7	45.7	2011/11/1	-46.0	-6.91
UGW6	32.75983	130.72788	22.3-33.3	35.3	2010/10/20	-47.1	-7.07
UGW6	32.75983	130.72788	22.3-33.3	35.3	2010/12/27	-45.7	-7.14

UGW6	32.75983	130.72788	22.3-33.3	35.3	2011/11/1	-46.4	-6.83
UGW7	32.76674	130.62894	6.75-12.3	15.0	2011/2/14	-41.6	-6.59
UGW8	32.76678	130.71235	34.7-51.2	80.0	2010/10/20	-47.1	-7.12
UGW8	32.76678	130.71235	34.7-51.2	80.0	2011/1/7	-45.0	-7.13
UGW8	32.76678	130.71235	34.7-51.2	80.0	2011/10/31	-46.2	-6.83
UGW9	32.76789	130.71928	9.6-15	15.0	2009/11/5	-47.7	-7.13
UGW9	32.76789	130.71928	9.6-15	15.0	2011/11/8	-45.9	-7.08
UGW10	32.76925	130.63753	9.5-12.5	15.0	2009/11/6	-45.4	-6.78
UGW11	32.77339	130.68631	10.0-15.0	15.0	2009/11/5	-45.5	-6.85
UGW11	32.77339	130.68631	10.0-15.0	15.0	2011/11/8	-45.1	-6.97
UGW12	32.80015	130.71570	10.4-15.9	21.4	2010/10/20	-48.1	-7.21
UGW12	32.80015	130.71570	10.4-15.9	21.4	2010/12/27	-46.7	-7.16
UGW13	32.81583	130.76250	19-30	30.0	2009/11/6	-46.1	-6.77
UGW13	32.81583	130.76250	19-30	30.0	2011/11/8	-45.2	-6.99
UGW14	32.81806	130.71444	14.2-25	25.0	2009/11/5	-46.4	-6.76
UGW14	32.81806	130.71444	14.2-25	25.0	2011/11/8	-44.8	-6.92
UGW15	32.82118	130.69851	15.5-26.5	32.0	2010/10/21	-47.8	-7.33
UGW15	32.82118	130.69851	15.5-26.5	32.0	2011/3/8	-47.2	-7.51
UGW15	32.82118	130.69851	15.5-26.5	32.0	2011/11/1	-50.0	-7.30
UGW16	32.83225	130.72431		10.0	2011/1/31	-45.1	-7.02
UGW17	32.84453	130.72338		7.6	2011/1/31	-45.2	-6.91
UGW17	32.84453	130.72338		7.6	2011/10/27	-46.7	-6.89
UGW18	32.84979	130.71870		9.6	2011/1/31	-46.0	-6.80
UGW18	32.84979	130.71870		9.6	2011/10/27	-46.8	-6.79
UGW19	32.86994	130.70298	54-59.5	60.0	2010/10/21	-44.6	-6.52
UGW19	32.86994	130.70298	54-59.5	60.0	2011/3/8	-42.7	-6.69
UGW19	32.86994	130.70298	54-59.5	60.0	2011/11/1	-45.4	-6.40
UGW20	32.77501	130.74613		24.8	2011/2/22	-47.3	-7.17
UGW20	32.77501	130.74613		24.8	2011/11/1	-46.8	-7.02
UGW21	32.77501	130.74613		25.0	2011/2/22	-46.6	-7.19
UGW22	32.78500	130.73519	5-10.5	16.0	2011/11/4	-46.2	-7.09
UGW23	32.78987	130.74242	33.7-46.5	46.5	2011/11/4	-46.4	-6.90

After the earthquake

Sample ID	Longitude	Latitude	Screen Depth (m)	Well Depth (m)	Sampling Day	δD_{H2O} (‰)	$\delta^{18}O_{H2O}$ (‰)
UGW1	32.73369	130.63492	4.5-10	10.0	2016/8/29	-37.9	-6.06

UGW1	32.73369	130.63492	4.5-10	10.0	2016/10/31	-37.9	-6.10
UGW1	32.73369	130.63492	4.5-10	10.0	2017/4/9	-37.7	-6.03
UGW1	32.73369	130.63492	4.5-10	10.0	2017/11/26	-37.2	-6.01
UGW2	32.73389	130.63639	5.0-10	10.0	2017/5/15	-40.6	-6.39
UGW3	32.74794	130.71631	24.7-35.7	41.2	2016/7/10	-46.2	-7.16
UGW3	32.74794	130.71631	24.7-35.7	41.2	2016/11/4	-46.6	-7.14
UGW3	32.74794	130.71631	24.7-35.7	41.2	2017/3/16	-46.4	-7.12
UGW3	32.74794	130.71631	24.7-35.7	41.2	2017/11/26	-46.6	-7.12
UGW4	32.75806	130.61111	7.0-11	15.0	2017/5/15	-44.4	-6.88
UGW5	32.75703	130.68156	31.7-42.7	45.7	2016/7/10	-45.6	-7.15
UGW5	32.75703	130.68156	31.7-42.7	45.7	2016/10/31	-46.5	-7.14
UGW5	32.75703	130.68156	31.7-42.7	45.7	2017/4/9	-46.0	-7.21
UGW5	32.75703	130.68156	31.7-42.7	45.7	2017/11/26	-46.3	-7.09
UGW6	32.75889	130.72708	22.3-33.3	35.3	2016/7/2	-47.1	-7.30
UGW6	32.75889	130.72708	22.3-33.3	35.3	2016/8/29	-46.8	-7.39
UGW6	32.75889	130.72708	22.3-33.3	35.3	2016/10/31	-46.8	-7.32
UGW6	32.75889	130.72708	22.3-33.3	35.3	2017/3/16	-46.9	-7.36
UGW6	32.75889	130.72708	22.3-33.3	35.3	2017/11/26	-46.5	-7.07
UGW7	32.76631	130.62967	6.75-12.3	15.0	2016/8/29	-42.6	-6.69
UGW7	32.76631	130.62967	6.75-12.3	15.0	2016/11/7	-42.9	-6.60
UGW7	32.76631	130.62967	6.75-12.3	15.0	2017/4/9	-43.3	-6.67
UGW7	32.76674	130.62894	6.75-12.3	15.0	2017/12/2	-43.2	-6.68
UGW8	32.76664	130.71203	34.7-51.2	80.0	2016/7/2	-46.0	-6.93
UGW8	32.76664	130.71203	34.7-51.2	80.0	2016/10/31	-46.9	-7.24
UGW8	32.76664	130.71203	34.7-51.2	80.0	2017/3/16	-47.1	-7.19
UGW8	32.76664	130.71203	34.7-51.2	80.0	2017/11/26	-46.5	-7.30
UGW9	32.77139	130.71694	9.6-15	15.0	2017/5/16	-45.9	-7.15
UGW10	32.77250	130.63528	9.5-12.5	15.0	2017/5/15	-43.4	-6.92
UGW11	32.77694	130.68389	10.0-15.0	15.0	2017/5/16	-47.2	-7.21
UGW12	32.79956	130.71619	10.4-15.9	21.4	2016/6/25	-47.5	-7.41
UGW12	32.79956	130.71619	10.4-15.9	21.4	2016/8/29	-47.7	-7.46
UGW12	32.79956	130.71619	10.4-15.9	21.4	2016/11/7	-47.4	-7.33
UGW12	32.79956	130.71619	10.4-15.9	21.4	2017/3/16	-46.4	-7.21
UGW12	32.79956	130.71619	10.4-15.9	21.4	2017/11/26	-46.6	-7.23
UGW13	32.81583	130.76250	19-30	30.0	2017/5/16	-45.4	-7.10
UGW14	32.81806	130.71444	14.2-25	25.0	2017/5/16	-45.0	-6.96
UGW15	32.82281	130.69800	15.5-26.5	32.0	2016/7/10	-47.5	-7.51

UGW15	32.82281	130.69800	15.5-26.5	32.0	2016/11/7	-47.7	-7.55
UGW15	32.82281	130.69800	15.5-26.5	32.0	2017/3/27	-46.3	-7.42
UGW15	32.82281	130.69800	15.5-26.5	32.0	2017/11/26	-47.1	-7.37
UGW16	32.83225	130.72431		10.0	2017/5/22	-46.3	-6.98
UGW19	32.86961	130.70300	54-59.5	60.0	2016/8/29	-45.8	-7.19
UGW19	32.86961	130.70300	54-59.5	60.0	2016/11/7	-46.2	-6.84
UGW19	32.86961	130.70300	54-59.5	60.0	2017/3/27	-44.6	-6.82
UGW19	32.86961	130.70300	54-59.5	60.0	2017/11/26	-44.5	-6.79
UGW20	32.84453	130.72338			2017/5/22	-45.2	-6.80
UGW21	32.84979	130.71870			2017/5/22	-44.8	-6.80

Supplementary Table 1e. Properties, chemistry, and oxygen and hydrogen stable isotope ratios (δD and $\delta^{18}O$) for confined groundwater samples used in this study.

Before the earthquake

Sample ID	Longitude	Latitude	Screen Depth (m)	Well Depth (m)	Sampling Day	δD_{H2O} (‰)	$\delta^{18}O_{H2O}$ (‰)
CGW1	32.729756	130.631155	112-145	145	2010/10/20	-45.3	-6.80
CGW1	32.72976	130.63116	112-145	145	2011/10/31	-45.1	-6.81
CGW1	32.72976	130.63116	112-145	145	2011/2/14	-43.5	-6.93
CGW2	32.72976	130.63116	53.2-75.2	93	2010/10/20	-45.4	-6.93
CGW2	32.72976	130.63116	53.2-75.2	93	2011/2/14	-44.5	-6.88
CGW2	32.72976	130.63116	53.2-75.2	93	2011/10/31	-43.9	-6.65
CGW3	32.73361	130.63583	50-60	60	2009/11/6	-47.9	-7.11
CGW4	32.73361	130.63583	90-105	120	2009/11/6	-43.9	-6.55
CGW5	32.74793	130.71634	95.3-106.3	112	2010/10/20	-47.7	-7.31
CGW5	32.74793	130.71634	95.3-106.3	112	2010/12/27	-46.4	-7.26
CGW5	32.74793	130.71634	95.3-106.3	112	2011/11/1	-46.7	-7.08
CGW6	32.74804	130.68876		200	2011/1/11	-47.2	-7.20
CGW6	32.74804	130.68876		200	2011/10/27	-47.9	-7.41
CGW7	32.75703	130.68156	121.5-138	155	2010/10/20	-46.7	-6.92
CGW7	32.75703	130.68156	121.5-138	155	2011/2/14	-47.2	-7.21
CGW7	32.75703	130.68156	121.5-138	155	2011/11/1	-46.6	-7.04
CGW8	32.75775	130.65308	113-129.5	135	2010/10/20	-47.0	-7.08
CGW8	32.75775	130.65308	113-129.5	135	2011/2/14	-46.6	-7.16
CGW9	32.75775	130.65308	71.9-93.7	109	2010/10/20	-47.2	-7.10

CGW9	32.75775	130.65308	71.9-93.7	109	2011/2/14	-46.4	-7.18
CGW10	32.75806	130.61111	60.5-77	80	2009/11/6	-46.5	-6.91
CGW11	32.75983	130.72788	97-108	110	2010/10/20	-47.6	-7.17
CGW11	32.75983	130.72788	97-108	110	2010/12/27	-46.7	-7.25
CGW11	32.75983	130.72788	97-108	110	2011/11/1	-47.4	-7.25
CGW12	32.76324	130.76213		211	2011/1/7	-47.2	-7.45
CGW13	32.76324	130.76213		130	2011/1/7	-47.2	-7.41
CGW13	32.76324	130.76213		130	2011/10/27	-48.0	-7.28
CGW14	32.76664	130.71203	82.32-98.82	115	2010/10/20	-46.8	-7.08
CGW14	32.76664	130.71203	82.32-98.82	115	2011/1/7	-46.3	-7.25
CGW14	32.76664	130.71203	82.32-98.82	115	2011/10/31	-47.6	-6.94
CGW15	32.76674	130.62894	51.4-67.9	100	2010/10/20	-47.5	-7.06
CGW15	32.76674	130.62894	51.4-67.9	100	2011/2/14	-46.4	-7.08
CGW16	32.76789	130.71928	45-60	50	2009/11/5	-48.0	-7.02
CGW16	32.76789	130.71928	45-60	50	2011/11/8	-46.1	-7.10
CGW17	32.76925	130.63753	58-74.5	80	2009/11/6	-47.9	-7.29
CGW18	32.77339	130.68631	45-65	65	2009/11/5	-47.4	-7.15
CGW18	32.77339	130.68631	45-65	65	2011/11/8	-46.1	-7.24
CGW19	32.77827	130.74330		41	2011/1/7	-46.7	-7.28
CGW20	32.77827	130.74330		44	2011/1/7	-46.7	-7.25
CGW20	32.77827	130.74330		44	2011/10/27	-47.3	-7.08
CGW21	32.78111	130.77722	45-55	55	2009/11/5	-49.0	-7.37
CGW21	32.78111	130.77722	45-55	55	2011/11/8	-47.5	-7.35
CGW22	32.78225	130.65788		120	2011/1/11	-46.2	-7.31
CGW23	32.78481	130.74818		45	2011/1/7	-46.6	-7.22
CGW23	32.78481	130.74818		45	2011/10/27	-47.2	-7.20
CGW24	32.79294	130.73953	39-50	55	2010/10/21	-47.2	-7.09
CGW24	32.79294	130.73953	39-50	55	2010/12/27	-46.3	-7.16
CGW25	32.79325	130.77711	48-59	71	2010/10/21	-47.2	-6.94
CGW25	32.79325	130.77711	48-59	71	2011/1/12	-46.2	-7.26
CGW25	32.79325	130.77711	48-59	71	2011/10/31	-49.6	-7.04
CGW26	32.79891	130.67285		120	2011/1/11	-46.8	-7.39
CGW27	32.79984	130.82750	58.5-80	80	2009/11/17	-44.0	-6.27
CGW27	32.79984	130.82750	58.5-80	80	2011/11/4	-42.5	-6.06
CGW28	32.79956	130.71619	39.5-55.4	65	2010/10/20	-47.9	-7.32
CGW28	32.79956	130.71619	39.5-55.4	65	2010/12/27	-47.7	-7.49
CGW28	32.79956	130.71619	39.5-55.4	65	2011/11/1	-48.4	-7.32

CGW29	32.80525	130.77051	120.5-131.5	137	2010/10/21	-46.7	-6.99
CGW29	32.80525	130.77051	120.5-131.5	137	2011/1/12	-46.1	-7.06
CGW29	32.80525	130.77051	120.5-131.5	137	2011/10/31	-49.3	-7.05
CGW30	32.81533	130.71650	34.2-45	50	2009/11/5	-47.1	-6.81
CGW30	32.81533	130.71650	34.2-45	50	2011/11/8	-45.3	-6.87
CGW31	32.81577	130.75250	47-58	60	2009/11/6	-46.2	-6.88
CGW31	32.81577	130.75250	47-58	60	2011/11/8	-45.4	-6.90
CGW32	32.81703	130.81222	65-70	70	2011/1/12	-46.6	-7.21
CGW32	32.81703	130.81222	65-70	70	2011/10/31	-49.7	-7.05
CGW33	32.81866	130.81912	60-90	90	2010/10/21	-49.7	-7.09
CGW33	32.81866	130.81912	60-90	90	2011/1/12	-46.0	-7.29
CGW33	32.81866	130.81912	60-90	90	2011/10/31	-49.1	-7.07
CGW34	32.82056	130.82167	23.5-83.5	90	2009/11/5	-48.9	-7.21
CGW34	32.82056	130.82167	23.5-83.5	90	2011/11/9	-47.0	-7.23
CGW35	32.82118	130.69851	59-75.5	81	2010/10/21	-47.2	-7.25
CGW35	32.82118	130.69851	59-75.5	81	2011/3/8	-46.8	-7.52
CGW35	32.82118	130.69851	59-75.5	81	2011/11/1	-49.7	-7.31
CGW36	32.82961	130.78981		90	2011/1/12	-44.9	-6.74
CGW36	32.82961	130.78981		90	2011/10/27	-45.9	-6.93
CGW37	32.83396	130.77102	71.39-93.45	110	2010/10/21	-45.7	-6.77
CGW37	32.83396	130.77102	71.39-93.45	110	2011/1/12	-44.3	-6.89
CGW37	32.83396	130.77102	71.39-93.45	110	2011/10/31	-47.4	-6.75
CGW38	32.83488	130.64478		140	2011/1/12	-46.7	-7.48
CGW39	32.84453	130.72338		131	2011/1/31	-46.7	-6.96
CGW39	32.84453	130.72338		131	2011/10/27	-47.1	-6.93
CGW40	32.84453	130.71776		124	2011/1/31	-46.9	-7.17
CGW40	32.84453	130.71776		124	2011/10/27	-47.4	-7.09
CGW41	32.84500	130.82153	81.2-86.7/117.8-140	140	2009/11/16	-45.3	-6.67
CGW41	32.84500	130.82153	81.2-86.7/117.8-140	140	2011/10/4	-44.7	-6.59
CGW42	32.84979	130.71870		100	2011/1/31	-47.4	-6.95
CGW43	32.85417	130.74490		121	2011/1/11	-46.4	-7.04
CGW44	32.85417	130.74490		145	2011/1/11	-45.3	-6.96
CGW44	32.85417	130.74490		145	2011/10/27	-45.5	-6.69
CGW45	32.85944	130.70654		151	2011/2/1	-44.4	-6.73
CGW46	32.85945	130.87016	96-116	120	2009/11/5	-49.3	-7.52
CGW46	32.85945	130.87016	96-116	120	2011/11/8	-48.5	-7.49
CGW47	32.86161	130.82744	88-98	103	2009/11/6	-48.4	-7.10

CGW47	32.86161	130.82744	88-98	103	2011/11/9	-47.0	-7.14
CGW48	32.86176	130.73567	88-98	101	2009/11/6	-44.9	-6.82
CGW48	32.86176	130.73567	88-98	101	2011/11/9	-44.5	-6.80
CGW49	32.86207	130.71963		152	2011/2/1	-44.8	-6.67
CGW50	32.86994	130.70298	83.5-94.5	100	2010/10/21	-46.5	-6.95
CGW50	32.86994	130.70298	83.5-94.5	100	2011/3/8	-45.5	-7.06
CGW50	32.86994	130.70298	83.5-94.5	100	2011/11/1	-47.5	-6.78
CGW51	32.87974	130.67590	51.5-91.5	95	2009/11/16	-44.5	-6.43
CGW51	32.87974	130.67590	51.5-91.5	95	2011/11/4	-47.2	-7.09
CGW52	32.89189	130.69344		150	2011/2/1	-45.8	-6.87
CGW52	32.89189	130.69344		150	2011/10/27	-46.2	-6.88
CGW53	32.89507	130.79147	81.5-98/102.5-114.5	121	2009/11/16	-42.9	-6.19
CGW53	32.89507	130.79147	81.5-98/102.5-114.5	121	2011/10/4	-45.4	-6.63
CGW54	32.89914	130.74418	43-70.5	82	2009/11/16	-47.1	-7.03
CGW54	32.89914	130.74418	43-70.5	82	2011/10/4	-44.6	-6.58
CGW55	32.90417	130.68595			2011/2/1	-46.5	-7.18
CGW55	32.90417	130.68595			2011/10/27	-47.0	-7.24
CGW56	32.90487	130.88368	80-118	120	2009/11/16	-44.4	-6.51
CGW57	32.92874	130.69999			2011/1/31	-46.6	-7.45
CGW58	32.69853	130.74818			2011/2/28	-45.0	-6.87
CGW59	32.71618	130.69938		150	2011/2/28	-47.3	-7.08
CGW59	32.71618	130.69938		150	2011/10/27	-46.3	-7.20
CGW60	32.71749	130.74330			2011/2/28	-44.3	-6.94
CGW60	32.71749	130.74330			2011/10/27	-45.2	-7.17
CGW61	32.72630	130.71412	72.5-83.5/89-111	117	2009/11/17	-48.2	-7.20
CGW61	32.72630	130.71412	72.5-83.5/89-111	117	2011/10/4	-48.1	-7.39
CGW62	32.70899	130.69171		50	2011/2/28	-46.9	-7.22
CGW63	32.71422	130.71472			2011/2/28	-49.7	-7.54
CGW64	32.71821	130.68034		160	2011/2/28	-49.1	-7.50
CGW65	32.72925	130.75097			2011/2/28	-42.6	-6.50
CGW66	32.74106	130.74128	29.5-46/51.5-57	60	2011/10/4	-49.6	-7.58
CGW67	32.76674	130.62894	127.5-149.5	210	2011/2/14	-44.9	-7.05
CGW68	32.77631	130.77677			2011/1/7	-47.0	-7.30
CGW69	32.77631	130.77677		207	2011/1/7	-47.2	-7.40
CGW69	32.77631	130.77677		207	2011/10/27	-48.1	-7.40
CGW70	32.78823	130.70738	44-55	55	2010/10/20	-44.9	-6.94
CGW71	32.84453	130.69999		152	2011/2/1	-45.6	-7.04

CGW72	32.87962	130.66817			2011/2/1	-46.2	-7.19
CGW72	32.87962	130.66817			2011/10/27	-44.6	-6.65
CGW73	32.88194	130.78443	58.5-69.5	75	2009/11/6	-47.3	-6.98
CGW73	32.88194	130.78443	58.5-69.5	75	2011/11/9	-45.5	-7.00

After the earthquake

Sample ID	Longitude	Latitude	Screen Depth (m)	Well Depth (m)	Sampling Day	δD_{H2O} (‰)	$\delta^{18}O_{H2O}$ (‰)
CGW1	32.72976	130.63116	112-145	145	2017/11/26	-44.7	-6.96
CGW2	32.72976	130.63116	53.2-75.2	93	2017/11/26	-46.5	-7.12
CGW3	32.73389	130.63639	50-60	60	2017/5/15	-46.6	-7.31
CGW4	32.73389	130.63639	90-105	120	2017/5/15	-45.2	-7.08
CGW5	32.74794	130.71631	95.3-106.3	112	2016/7/2	-47.4	-7.43
CGW5	32.74794	130.71631	95.3-106.3	112	2016/10/31	-47.4	-7.36
CGW5	32.74794	130.71631	95.3-106.3	112	2017/3/16	-47.0	-7.22
CGW5	32.74794	130.71631	95.3-106.3	112	2017/11/26	-47.2	-7.40
CGW6	32.74804	130.68876		200	2017/5/23	-47.9	-7.44
CGW7	32.75703	130.68156	121.5-138	155	2016/7/10	-46.8	-7.28
CGW7	32.75703	130.68156	121.5-138	155	2017/4/9	-46.7	-7.13
CGW7	32.75703	130.68156	121.5-138	155	2016/10/31	-47.1	-7.21
CGW8	32.75778	130.65306	113-129.5	135	2016/7/2	-46.8	-7.33
CGW8	32.75778	130.65306	113-129.5	135	2017/4/9	-47.2	-7.32
CGW8	32.75778	130.65306	113-129.5	135	2016/10/31	-46.8	-7.26
CGW8	32.75778	130.65306	113-129.5	135	2017/11/26	-46.9	-7.33
CGW9	32.75778	130.65306	71.9-93.7	109	2016/7/2	-46.9	-7.36
CGW9	32.75778	130.65306	71.9-93.7	109	2016/10/31	-47.3	-7.25
CGW9	32.75778	130.65306	71.9-93.7	109	2017/4/9	-46.6	-7.27
CGW9	32.75778	130.65306	71.9-93.7	109	2017/11/26	-46.8	-7.44
CGW10	32.75806	130.61111	60.5-77	80	2017/5/15	-44.4	-6.72
CGW11	32.75889	130.72708	97-108	110	2016/8/29	-47.4	-7.43
CGW11	32.75889	130.72708	97-108	110	2016/10/31	-47.3	-7.40
CGW11	32.75889	130.72708	97-108	110	2016/7/2	-47.3	-7.33
CGW11	32.75889	130.72708	97-108	110	2017/3/16	-47.0	-7.22
CGW11	32.75889	130.72708	97-108	110	2017/11/26	-47.3	-7.30
CGW12	32.76324	130.76213		211	2017/5/8	-48.5	-7.58
CGW13	32.76324	130.76213		130	2017/5/8	-48.2	-7.59
CGW14	32.76664	130.71203	82.32-98.82	115	2016/10/31	-47.1	-7.28

CGW14	32.76664	130.71203	82.32-98.82	115	2016/7/2	-46.8	-7.26
CGW14	32.76664	130.71203	82.32-98.82	115	2017/3/16	-45.8	-7.20
CGW15	32.76674	130.62894	51.4-67.9	100	2017/12/2	-45.2	-6.99
CGW16	32.77139	130.71694	45-60	50	2017/5/16	-46.6	-7.24
CGW17	32.77250	130.63528	58-74.5	80	2017/5/15	-46.8	-7.42
CGW18	32.77694	130.68389	45-65	65	2017/5/16	-44.2	-6.84
CGW19	32.77828	130.74330		41	2017/5/8	-47.5	-7.47
CGW20	32.77828	130.74330		44	2017/5/8	-47.1	-7.37
CGW21	32.78111	130.77722	45-55	55	2017/5/15	-48.2	-7.49
CGW22	32.78225	130.65788		120	2017/5/23	-47.0	-7.31
CGW23	32.78481	130.74818		45	2017/5/9	-47.1	-7.31
CGW24	32.79294	130.73953	39-50	55	2017/3/16	-47.0	-7.36
CGW24	32.79294	130.73953	39-50	55	2016/6/25	-46.9	-7.33
CGW24	32.79294	130.73953	39-50	55	2016/11/7	-47.0	-7.32
CGW24	32.79294	130.73953	39-50	55	2017/11/26	-48.1	-7.33
CGW25	32.79325	130.77711	48-59	71	2016/10/31	-47.3	-7.41
CGW25	32.79325	130.77711	48-59	71	2016/8/29	-47.3	-7.32
CGW25	32.79325	130.77711	48-59	71	2016/6/25	-46.8	-7.30
CGW25	32.79325	130.77711	48-59	71	2017/3/16	-47.2	-7.24
CGW25	32.79325	130.77711	48-59	71	2017/11/26	-47.2	-7.29
CGW26	32.79891	130.67285		120	2017/5/23	-46.9	-7.40
CGW27	32.79984	130.82750	58.5-80	80	2017/5/1	-45.5	-6.88
CGW28	32.79956	130.71619	39.5-55.4	65	2016/8/29	-48.3	-7.63
CGW28	32.79956	130.71619	39.5-55.4	65	2017/4/9	-48.3	-7.60
CGW28	32.79956	130.71619	39.5-55.4	65	2016/11/7	-48.6	-7.57
CGW28	32.79956	130.71619	39.5-55.4	65	2016/6/25	-48.0	-7.54
CGW28	32.79956	130.71619	39.5-55.4	65	2017/3/16	-48.0	-7.49
CGW28	32.79956	130.71619	39.5-55.4	65	2017/11/26	-48.0	-7.43
CGW29	32.80489	130.76942	120.5-131.5	137	2016/10/31	-46.9	-7.20
CGW29	32.80489	130.76942	120.5-131.5	137	2017/3/16	-46.6	-7.17
CGW29	32.80489	130.76942	120.5-131.5	137	2016/6/25	-46.5	-7.12
CGW29	32.80489	130.76942	120.5-131.5	137	2017/11/26	-46.4	-7.33
CGW30	32.81806	130.71444	34.2-45	50	2017/5/16	-46.0	-7.11
CGW31	32.81583	130.76250	47-58	60	2017/5/16	-45.7	-6.94
CGW32	32.81714	130.81231	65-70	70	2016/6/25	-47.1	-7.32
CGW32	32.81714	130.81231	65-70	70	2016/10/31	-47.4	-7.39
CGW32	32.81714	130.81231	65-70	70	2017/4/9	-47.3	-7.38

CGW32	32.81714	130.81231	65-70	70	2017/3/16	-47.3	-7.38
CGW32	32.81714	130.81231	65-70	70	2017/11/26	-47.2	-7.35
CGW33	32.81861	130.81906	60-90	90	2016/8/29	-47.3	-7.45
CGW33	32.81861	130.81906	60-90	90	2016/10/31	-47.4	-7.41
CGW33	32.81861	130.81906	60-90	90	2016/6/25	-47.4	-7.33
CGW33	32.81861	130.81906	60-90	90	2017/3/16	-46.5	-7.32
CGW33	32.81861	130.81906	60-90	90	2017/11/26	-47.0	-7.38
CGW34	32.82056	130.82167	23.5-83.5	90	2017/5/16	-47.3	-7.23
CGW35	32.82281	130.69800	59-75.5	81	2016/11/7	-47.6	-7.56
CGW35	32.82281	130.69800	59-75.5	81	2016/7/10	-47.4	-7.52
CGW35	32.82281	130.69800	59-75.5	81	2017/3/27	-47.7	-7.52
CGW35	32.82281	130.69800	59-75.5	81	2017/11/26	-47.6	-7.49
CGW36	32.82961	130.78981		90	2017/5/9	-45.3	-7.00
CGW37	32.83439	130.77128	71.39-93.45	110	2016/10/31	-45.4	-7.02
CGW37	32.83439	130.77128	71.39-93.45	110	2017/3/16	-45.6	-7.00
CGW37	32.83439	130.77128	71.39-93.45	110	2016/6/25	-45.3	-6.89
CGW37	32.83439	130.77128	71.39-93.45	110	2017/11/26	-43.9	-6.57
CGW38	32.83488	130.64478		140	2017/5/23	-47.3	-7.47
CGW39	32.84453	130.72338		131	2017/5/22	-47.2	-7.23
CGW40	32.84453	130.71776		124	2017/5/22	-47.2	-7.47
CGW41	32.84476	130.82150	81.2-86.7/117.8-140	140	2017/5/1	-46.5	-7.22
CGW42	32.84979	130.71870		100	2017/5/22	-46.8	-7.27
CGW43	32.85417	130.74490		121	2017/5/22	-45.7	-7.21
CGW44	32.85417	130.74490		145	2017/5/22	-46.3	-7.21
CGW45	32.85944	130.70654		151	2017/5/22	-45.8	-6.91
CGW46	32.85972	130.86944	96-116	120	2017/5/19	-49.1	-7.65
CGW47	32.86056	130.82806	88-98	103	2017/5/16	-47.1	-7.15
CGW48	32.86139	130.73500	88-98	101	2017/5/16	-47.7	-7.28
CGW49	32.86207	130.71963		152	2017/5/24	-45.6	-6.88
CGW50	32.86961	130.70300	83.5-94.5	100	2016/8/29	-46.1	-7.07
CGW50	32.86961	130.70300	83.5-94.5	100	2016/11/7	-46.5	-7.05
CGW50	32.86961	130.70300	83.5-94.5	100	2017/3/27	-46.3	-7.00
CGW50	32.86961	130.70300	83.5-94.5	100	2017/11/26	-45.3	-6.86
CGW51	32.87965	130.67580	51.5-91.5	95	2017/5/1	-46.4	-7.07
CGW52	32.89189	130.69344		150	2017/5/24	-45.7	-6.90
CGW53	32.89526	130.79138	81.5-98/102.5-114.5	121	2017/4/26	-46.4	-7.19
CGW54	32.89918	130.74428	43-70.5	82	2017/4/26	-47.1	-7.31

CGW55	32.90417	130.68595			2017/5/24	-46.4	-7.28
CGW56	32.90485	130.88378	80-118	120	2017/4/26	-45.5	-7.20
CGW57	32.92874	130.69999			2017/5/24	-47.3	-7.59
CGW58	32.69853	130.74818			2017/5/23	-44.5	-7.06
CGW59	32.71618	130.69938		150	2017/5/23	-45.8	-7.34
CGW60	32.71749	130.74330			2017/5/23	-46.5	-7.39
CGW61	32.72640	130.71480	72.5-83.5/89-111	117	2017/5/10	-47.8	-7.50
CGW74	32.83225	130.72431			2017/5/22	-45.5	-7.21
CGW75	32.86796	130.70264			2017/5/24	-46.0	-6.99

Supplementary Table 1f. Properties, chemistry, and oxygen and hydrogen stable isotope ratios (δD and $\delta^{18}O$) for hot spring water samples used in this study.

**After the earthquake**						
Sample ID	Latitude	Longitude	Sampling Day	Depth	δD_{H2O} (‰)	$\delta^{18}O_{H2O}$ (‰)
HS18001	32.810833	130.701389	2018/8/8	1000	-49.9	-7.63
HS18002	32.785375	130.702819	2018/7/24	1000	-48.7	-7.36
HS18003	32.736058	130.748169	2018/7/24	1000	-36.5	-1.84
HS18004	32.714369	130.811675	2018/7/24	1000	-54.1	-8.21
HS18005	32.790317	130.776567	2018/7/24	1150	-46.5	-7.21
HS18006	32.876731	130.734283	2018/7/8	200	-47.1	-6.75
HS18007	32.873531	130.728942	2018/7/8	180	-47.6	-6.94
HS18008	32.885914	130.730881	2018/7/8	300	-47.5	-6.86
HS18009	32.935261	130.725189	2018/7/8	1000	-52.7	-5.53
HS18010	32.933431	130.802672	2018/7/10	1113	-58.4	-7.06
HS18011	32.842414	130.781722	2018/7/8	1200	-45.5	-7.17
HS18013	32.892922	130.842056	2018/7/8	1000	-51.5	-3.94
HS18014	32.876218	130.864785	2018/7/24	?	-52.8	-3.59
HS18015	32.835864	131.069183	2018/7/10	1300	-55.9	-8.67
HS18016	32.844903	131.045197	2018/7/10	1000	-56.3	-8.88
HS18017	32.871447	131.005906	2018/7/10	800	-54.7	-8.51
HS18018	32.904858	130.989819	2018/7/10	1000	-54.7	-8.44
HS18019	32.977720	131.042790	2018/7/10	200	-53.3	-8.43
HS18020	32.931389	130.774722	2018/7/10	700	-54.4	-5.02
HS18021	32.932222	130.739167	2018/8/8	500	-51.3	-5.64
HS18022	32.956567	130.723847	2018/8/8	350	-47.6	-6.95
HS18023	32.962167	130.750381	2018/8/8	993	-52.5	-6.31
HS18024	32.982681	130.818786	2018/8/8	500	-50.7	-7.76

Supplementary Table 1g. Properties, chemistry, and oxygen and hydrogen stable isotope ratios (δD and $\delta^{18}O$) for mountain water sample obtained from tunnel.

**After the earthquake**					
Sample ID	Latitude	Longitude	Sampling Day	δD_{H2O} (‰)	$\delta^{18}O_{H2O}$ (‰)
Tunnel	32.93122	130.9366667	2017/10/7	-50.82	-8.00

Supplementary Table 2. Oxygen and hydrogen stable isotope ratios (δD and $\delta^{18}O$) for groundwater samples collected from cross-check sampling surveys.

Sample ID	Sampling day	Latitude		Longitude		δD_{H2O} (‰)	$\delta^{18}O_{H2O}$ (‰)
		**2015 sampling (before the earthquake)**					
T-11	2015/9/7	N	32.759832	E	130.727883	-47.7	-7.29
T-12	2015/9/7	N	32.818657	E	130.819120	-47.8	-7.28
T-21	2015/9/9	N	32.766738	E	130.628937	-42.9	-6.40
Shimoezu	2015/9/7	N	32.773086	E	130.749168	-47.9	-7.30
T-34	2015/9/7	N	32.800154	E	130.715702	-48.7	-7.42
T-35	2015/9/7	N	32.800154	E	130.715702	-48.5	-7.33
T-40	2015/9/7	N	32.793227	E	130.777260	-47.4	-7.19
T-41	2015/9/9	N	32.869939	E	130.702980	-44.9	-6.63
T-45	2015/9/9	N	32.729756	E	130.631155	-38.1	-5.83
M-209	2015/9/9	N	32.893737	E	130.710665	-45.0	-6.59
M-223	2015/9/9	N	32.865222	E	130.693492	-45.3	-6.81
M-229	2015/9/9	N	32.860659	E	130.712982	-46.7	-6.98
M-236	2015/9/9	N	32.886399	E	130.726962	-44.9	-6.55
M-217	2015/9/9	N	32.896003	E	130.708151	-43.4	-6.39
M-224	2015/9/9	N	32.862714	E	130.695053	-49.9	-7.36
M-242	2015/9/9	N	32.880015	E	130.698400	-46.9	-6.97
M33	2015/9/8	N	32.886074	E	130.765103	-46.8	-6.92
T-1	2015/9/8	N	32.872841	E	130.821262	-47.8	-7.05
M-236-2	2015/9/9	N	32.883029	E	130.715662	-47.7	-7.10
		**2016 sampling (after the earthquake)**					
T-21	2016/8/29	N	32.766297	E	130.629635	-43.0	-6.54
T-45	2016/8/29	N	32.733687	E	130.634919	-38.1	-5.89
T-40	2016/8/29	N	32.793187	E	130.777194	-47.4	-7.19
T-12	2016/8/29	N	32.818693	E	130.819407	-47.8	-7.27
T-10	2016/8/29	N	32.758655	E	130.727048	-47.4	-7.19
T-11	2016/8/29	N	32.758655	E	130.727048	-47.8	-7.29
Shimoezu	2016/8/29	N	32.773090	E	130.749112	-48.0	-7.31

T-35	2016/8/29	N	32.799581	E	130.716201	-48.1	-7.26
T-34	2016/8/29	N	32.799581	E	130.716201	-48.7	-7.42
T-41	2016/8/29	N	32.869626	E	130.703015	-44.7	-6.56
T-42	2016/8/29	N	32.869626	E	130.703015	-46.5	-6.90
			**2017 sampling (after the earthquake)**				
T-40	2017/3/16	N	32.793187	E	130.777194	-45.6	-6.74
T-12	2017/3/16	N	32.818693	E	130.819407	-48.0	-7.55
T-13	2017/3/16	N	32.817130	E	130.812300	-46.5	-7.06
T-36	2017/3/16	N	32.834380	E	130.771270	-45.0	-6.81
T-51	2017/3/16	N	32.804880	E	130.769410	-47.6	-7.43
T-9	2017/3/16	N	32.799550	E	130.739500	-47.1	-7.29
T-9-2	2017/3/16	N	32.799550	E	130.739500	-47.8	-7.49
T-35	2017/3/16	N	32.799581	E	130.716201	-46.7	-7.20
T-34	2017/3/16	N	32.799581	E	130.716201	-48.2	-7.48
T-43	2017/3/16	N	32.766630	E	130.712020	-47.0	-7.29
T-44	2017/3/16	N	32.766630	E	130.712020	-42.1	-5.94
T-10	2017/3/16	N	32.758655	E	130.727048	-47.1	-7.17
T-10-2	2017/3/16	N	32.758655	E	130.727048	-46.6	-7.10
T-11	2017/3/16	N	32.758655	E	130.727048	-49.1	-7.70
T-18	2017/3/16	N	32.747940	E	130.716300	-48.0	-7.46
T-18-2	2017/3/16	N	32.747940	E	130.716300	-47.1	-7.17
T-17	2017/3/16	N	32.747940	E	130.716300	-48.0	-7.34
T-14	2017/4/9	N	32.757020	E	130.681550	-47.8	-7.45
T-15	2017/4/9	N	32.757020	E	130.681550	-48.6	-7.57
T-49	2017/3/27	N	32.822800	E	130.697990	-49.0	-7.73
T-50	2017/3/27	N	32.822800	E	130.697990	-49.2	-7.82
T-41	2017/3/27	N	32.869611	E	130.703000	-46.4	-7.10
T-42	2017/3/27	N	32.869611	E	130.703000	-47.5	-7.24

Supplementary Table 3. Oxygen and hydrogen stable isotope ratios (δD and $\delta^{18}O$) for groundwater samples collected from discharge areas reported by ref. 5.

Site name	S1		S2		S3		S4		L1	
	δD_{H2O} (‰)	$\delta^{18}O_{H2O}$ (‰)	δD_{H2O} (‰)	$\delta^{18}O_{H2O}$ (‰)	δD_{H2O} (‰)	$\delta^{18}O_{H2O}$ (‰)	δD_{H2O} (‰)	$\delta^{18}O_{H2O}$ (‰)	δD_{H2O} (‰)	$\delta^{18}O_{H2O}$ (‰)
9-Jun-09	-47.3	-7.0	-47.3	-7.0	-47.5	-6.9	-48.1	-6.9	-7.0	-47.8
9-Jul-09	-47.7	-7.0	-47.7	-7.1	-47.3	-7.0	-48.3	-7.2	-7.2	-48.3
9-Aug-09	-47.2	-6.9	-47.5	-6.9	-47.9	-7.1	-47.9	-7.3	-7.2	-47.3
9-Sep-09	-47.8	-7.1	-47.8	-7.2	-47.7	-7.1	-48.1	-7.2	-7.2	-47.7
9-Oct-09	-47.5	-7.1	-47.7	-7.1	-48.0	-7.2	-48.0	-7.0	-7.1	-47.9
9-Nov-09	-47.5	-6.9	-47.9	-7.0	-47.9	-6.9	-48.4	-7.1	-6.9	-48.3
9-Dec-09	-47.3	-7.0	-47.4	-7.1	-47.8	-7.2	-48.1	-7.2	-7.1	-47.5
10-Jan-10	-47.4	-7.1	-47.8	-7.1	-47.9	-7.1	-47.9	-7.1	-7.1	-47.5
10-Feb-10	-48.2	-7.1	-47.5	-7.1	-47.4	-7.2	-47.9	-7.2	-7.1	-47.6
10-Mar-10	-47.5	-7.1	-47.9	-7.0	-48.1	-7.1	-47.8	-7.2	-7.1	-47.1
10-Apr-10	-47.6	-7.2	-47.9	-7.2	-47.7	-6.9	-48.8	-7.3	-7.2	-48.1
10-May-10	-48.0	-7.0	-48.3	-7.0	-48.3	-7.1	-48.8	-7.2	-7.1	-48.4
10-Jun-10	-48.3	-6.9	-48.3	-7.1	-48.3	-7.1	-49.3	-7.1	-6.9	-48.7
10-Jul-10	-47.2	-6.9	-48.6	-7.0	-47.0	-7.0	-47.4	-7.1	-7.3	-47.8
Standard deviation	0.35	0.10	0.37	0.08	0.37	0.11	0.49	0.11	0.11	0.46

Samples from the sites S1, S2 and S3 are waters from unconfined aquifers discharging in Lake Ezu, while those from S4 are artesian well waters from confined aquifers.

Samples in the site L1 are the Ezu Lake waters (groundwater fed lake). See Fig. 2 in ref. 5 for the location of each site.

Supplementary References

1. Hosono, T., Yamada, C., Shibata, T., Tawara, Y., Wang, C. -Y., Manga, M., Rahman, A. T. M. S. & Shimada, J. Coseismic groundwater drawdown along crustal ruptures during the 2016 M_w 7.0 Kumamoto earthquake. *Water Resour. Res.* **55**(7), 5891–5903 (2019).
2. Hosono, T., Tokunaga, T., Kagabu, M., Nakata, H., Orishikida, T., Lin, I. -T. & Shimada, J. The use of δ^{15}N and δ^{18}O tracers with an understanding of groundwater flow dynamics for evaluating the origins and attenuation mechanisms of nitrate pollution. *Water Res.* **47**, 2661-2675 (2013).
3. Tawara, Y., Hosono, T., Fukuoka, Y., Yoshida, T. & Shimada, J. Quantitative assessment of the changes in regional water flow systems caused by the 2016 Kumamoto Earthquake using numerical modeling. *J. Hydrol.* **583,** 124559 (2020).
4. Ide, K., Hosono, T., Kagabu, M., Fukamizu, K., Tokunaga, T. & Shimada, J. Changes of groundwater flow systems after the 2016 M_w 7.0 Kumamoto earthquake deduced by stable isotopic and CFC-12 compositions of natural springs. *J. Hydrol.* **583,** 124551 (2020).
5. Ono, M., Shimada, J., Ichikawa, T. & Tokunaga, T. Evaluation of groundwater discharge in Lake Ezu, Kumamoto, based on radon in water. *Jap. J. Limnol.* **72,** 193-210 (2011) (in Japanese with English abstract).
